# Spectral Properties of Brain Activity Under Two Anesthetics and Their Potential for Inducing Natural Sleep in Birds

**DOI:** 10.3389/fnins.2018.00881

**Published:** 2018-11-27

**Authors:** Ryan K. Tisdale, Laura Tieri, Niels C. Rattenborg, Gabriel J. L. Beckers, John A. Lesku

**Affiliations:** ^1^Avian Sleep Group, Max Planck Institute for Ornithology, Seewiesen, Germany; ^2^School of Life Sciences, La Trobe University, Melbourne, VIC, Australia; ^3^Cognitive Neurobiology and Helmholtz Institute, Utrecht University, Utrecht, Netherlands

**Keywords:** EEG, pigeon, isoflurane, urethane, slow wave sleep, burst suppression, unihemispheric, coherence

## Abstract

Both mammals and birds exhibit two sleep states, slow wave sleep (SWS) and rapid eye movement (REM) sleep. Studying certain aspects of sleep-related electrophysiology in freely behaving animals can present numerous methodological constraints, particularly when even fine body movements interfere with electrophysiological signals. Interestingly, under light general anesthesia, mammals and birds also exhibit slow waves similar to those observed during natural SWS. For these reasons, slow waves occurring under general anesthesia are commonly used in the investigation of sleep-related neurophysiology. However, how spectral properties of slow waves induced by anesthesia correspond to those occurring during natural SWS in birds has yet to be investigated systematically. In this study, we systematically analyzed spectral properties of electroencephalographic (EEG) patterns of pigeons (*Columba livia*) occurring under two commonly used anesthetics, isoflurane and urethane. These data were compared with EEG patterns during natural sleep. Slow waves occurring during spontaneous SWS, and those induced with isoflurane and urethane all showed greatest absolute power in the slowest frequencies (<3 Hz). Isoflurane and urethane-induced slow waves had near-identical power spectra, and both had higher mean power than that observed during SWS for all frequencies examined (0–25 Hz). Interestingly, burst suppression EEG activity observed under deeper planes of isoflurane anesthesia could occur bihemispherically or unihemispherically. Electrophysiological patterns while under isoflurane and urethane share phenomenological and spectral similarities to those occurring during SWS, notably the generation of high amplitude, slow waves, and peak low-frequency power. These results build upon other studies which suggest that some anesthetics exert their effects by acting on natural sleep pathways. As such, anesthesia-induced slow waves appear to provide an acceptable model for researchers interested in investigating sleep-related slow waves utilizing electrophysiological methods not suitable for use in freely behaving birds.

## Introduction

Sleep remains one of the great mysteries in the study of neuroscience. All animals studied have been found to sleep (Nath et al., 2017; Omond et al., 2017) and most spend a relatively large amount of time sleeping (Lesku et al., 2008), suggesting that sleep serves important functions. In spite of sleep’s conspicuous and ubiquitous presence across the animal kingdom, relatively little is known about the mechanisms underlying the generation of the brain rhythms associated with sleep outside of mammals.

The presence of two distinct sleep sub-states, slow wave sleep (SWS) and rapid eye movement (REM) sleep, in both mammals and birds suggests that sleep might serve multiple functions (Vyazovskiy and Delogu, 2014). In both mammals and birds, SWS is associated with stereotyped changes in electrophysiological data, notably the presence of high amplitude, low frequency (slow) waves in the forebrain electroencephalogram (EEG), typically characterized as 0.5–4 Hz power density (Steriade et al., 1993a,b,c; Rattenborg et al., 2011; Lesku and Rattenborg, 2014). REM sleep on the other hand is characterized by low amplitude, high frequency EEG activity similar to the patterns seen during wakefulness, occurring in conjunction with a number of behaviors, including closure of the eyes, head dropping resulting from reduced muscle tone, myoclonic twitches, and rapid eye movements (Dewasmes et al., 1985; Siegel, 2016).

Studying electrophysiology during natural sleep in animals presents certain experimental and technological constraints. The ability to pharmacologically induce the brain rhythms normally associated with sleep states is of great interest for electrophysiologists. Interestingly, a number of anesthetics induce brain rhythms similar to those observed during natural sleep in mammals, and are thus a frequently used model for the study of sleep-related brain rhythms (Steriade et al., 1993a,b,c; Amzica and Steriade, 1995; Chauvette et al., 2011; Beckers et al., 2014; Van Der Meij et al., 2015; Tisdale et al., 2018). Studies under anesthesia allow the use of recording methods not suitable for use in naturally sleeping animals, such as intracellular recordings of neuronal membrane potentials. However, in contrast to mammals, little is known about how anesthesia-induced states compare to natural sleep in birds.

Isoflurane, and other halogenated inhalant anesthetics, are commonly used to study sleep-related electrophysiology (Gugino et al., 2001; Mohajerani et al., 2010, 2013). Isoflurane-anesthetized mammals exhibit EEG slow waves, which share numerous attributes with naturally occurring SWS-related EEG slow waves. Following sleep deprivation, isoflurane anesthesia relieves homeostatic pressure for SWS as if natural sleep had occurred (Nelson et al., 2010). Isoflurane causes an increase in neuronal discharge in the ventrolateral preoptic nucleus of the hypothalamus, a key sleep-promoting region involved in the generation of natural sleep (Moore et al., 2012). Isoflurane has also recently been demonstrated to activate sleep centers in the fly brain (Kottler et al., 2013; Cohen et al., 2016; Zalucki and van Swinderen, 2016). This all suggests that not only do isoflurane-induced slow waves perform similar functional processes to naturally occurring slow waves, but also that isoflurane may exert its effect by acting on the same neuronal networks that initiate natural sleep. In addition, isoflurane may exert those effects in a conserved manner, from flies to mammals.

Under isoflurane, periods of EEG slow waves alternate with periods of isoelectric EEG patterns, which become longer and more frequent under higher levels of isoflurane (Kroeger and Amzica, 2007). Conversely, at low doses of isoflurane, suppression periods are nearly abolished, with the EEG being characterized by nearly continuous slow waves. These alternating EEG patterns have been termed burst suppression (Kroeger and Amzica, 2007). Burst suppression is also induced by other anesthetics, including ketamine-xylazine (Coenen et al., 2008).

Another anesthetic of particular interest for sleep researchers is urethane. Urethane anesthesia has been shown to induce alternating brainwave patterns resembling the SWS/REM sleep cycling that occurs during natural sleep in mammals (Clement et al., 2008). Brainwave patterns alternate between high amplitude, low frequency slow waves, and EEG activation occurring in conjunction with reduced nuchal muscle tone, and phasic eye and limb movements (Clement et al., 2008). The potential ability to induce a REM sleep-like state is unique to urethane among anesthetics, and is of particular interest to researchers studying REM sleep-related behaviors and neurophysiology.

Birds are of obvious interest for comparative studies given the presence of similar sleep states in both birds and mammals, and the absence of unequivocal evidence for similar sleep states in other phylogenetic groups (Libourel and Herrel, 2016; Shein-Idelson et al., 2016; Tisdale et al., 2018). Relatively few studies have investigated EEG patterns under isoflurane or urethane anesthesia in phylogenetic groups outside of mammals (Ookawa, 1967; Shibata and Kadono, 1970; Beckers et al., 2014; Tisdale et al., 2018). Under isoflurane anesthesia, zebra finches (*Taeniopygia guttata*) exhibit high amplitude, low frequency slow waves similar in form to those occurring during natural SWS (Beckers et al., 2014). In addition, brainwave patterns in isoflurane anesthetized zebra finches (Beckers et al., 2014) and halothane anesthetized chickens (*Gallus gallus domesticus*) (McIlhone et al., 2018) showed similar dose-dependent burst suppression patterns to those observed in anesthetized mammals. Finally, recordings from the hyperpallium of chickens anesthetized with urethane revealed periods of EEG slow waves, intermittently interrupted by periods of activation, which were associated with reduced neck muscle tone and heart rate, similar to the cyclic patterns observed in mammals, suggesting that urethane may be capable of inducing a similar REM sleep-like state in birds (Shibata and Kadono, 1970). Although these few studies suggest that birds respond similarly to mammals to anesthetics, the spectral properties of anesthetic-induced states have not been systematically compared to those occurring during natural sleep.

Here, we systematically evaluated anesthetic regiments commonly used in the study of sleep-related brain rhythms in mammals, in pigeons (*Columba livia*), a species often used in laboratory-based research. The spectral properties of brain rhythms occurring under isoflurane and urethane anesthesia were compared to the spectral properties of naturally occurring SWS recorded from freely behaving birds.

## Materials and Methods

### Implant Procedure

Anesthesia was induced using isoflurane (5% vaporized in 1.0 LPM O_2_) and a surgical plane was maintained using a lower dose (1.5–2.0% vaporized in 1.0 LPM O_2_). To detect sleep-related changes in brain activity, four EEG electrodes were implanted epidurally along a single row: two electrodes over each hemisphere, with two over the mesopallium, and two over the hyperpallium. As with other avian species, the hyperpallium could be seen through the cranium facilitating electrode placement. Hyperpallial electrodes were placed 2.0 mm to either side of the midline, while mesopallial electrodes were place 4.0 mm to either side of the midline. Recording electrodes were referenced to an electrode placed on the cerebellum. The electrodes were attached to a connector mounted on the bird’s head with dental acrylic. The pigeons were allowed at least 1-week post-operative recovery before recordings commenced.

### Natural Sleep Recordings

Pigeons (*n* = 7) were housed individually in enclosures measuring 79 cm length × 60 cm width × 60 cm height with a mesh window allowing ventilation. The back wall of the enclosure was composed of translucent Plexiglas and lights were mounted on the outside of this wall (400–500 lux at head level in the center of the box). Lights were maintained on a 12 h light/12 h dark photoperiod. A low, circular perch was provided in the center of the enclosure. Infrared sensitive cameras were placed in each corner and an infrared light source (940 nm) was mounted on the ceiling in the middle of the enclosure. The bird’s head plug was tethered to a ceiling-mounted commutator (Plastics One, Inc.^1^). Following at least 1-week of habituation to these recording conditions, baseline recordings were carried out, commencing at lights off and lasted the entirety of the 12 h night. Four unipolar EEG derivations referenced to the cerebellum were recorded at 200 Hz using a tethered recording system (Embla A10, Embla, Broomfield, CO, United States; data previously published in Lesku et al., 2011).

### Isoflurane Recordings

Four unipolar EEG derivations referenced to the cerebellum were recorded at 200 Hz using a tethered recording system (Embla N7000, Embla, Broomfield, CO, United States) for seven (other) birds anesthetized with isoflurane. Anesthesia was induced using isoflurane (5% vaporized in 1.0 LPM O_2_). Once a loss of consciousness was achieved, the level of isoflurane was lowered to 4% and a recording tether was attached to the bird, and the isoflurane level was then further lowered to 1.5%. Anesthesia depth was monitored and maintained at a low level (0.9–1.5% vaporized in 1.0 LPM O_2_) by testing pedal reflex response. If the bird showed signs of arousing (eye opening, postural changes), the level of isoflurane was acutely increased to 3–4%, followed by subsequent reductions in isoflurane concentration.

### Urethane Recordings

Four unipolar EEG derivations referenced to the cerebellum were recorded at 200 Hz using a tethered recording system (Embla N7000, Embla, Broomfield, CO, United States) in four pigeons anesthetized with urethane. Anesthesia was induced using isoflurane (5% vaporized in 1.0 LPM O_2_). The recording tether was connected once a surgical plane of anesthetic was reached. An intravenous (*n* = 3) or intraosseous (*n* = 1) access catheter was inserted into the right basilic vein or right femur, respectively. Once vascular access was established, isoflurane was discontinued and an initial dose of urethane was administered to each pigeon (0.1, 1.0, or 1.5 ml), followed by variable increments every 15–25 min for the duration of the multi-hour recording session. Final dosages of urethane did not exceed 3 ml. The pigeons were closely filmed throughout the urethane recordings to later scrutinize for the presence of eye movements. Following the recording session, isoflurane was re-administered (5% vaporized in 1.0 LPM O_2_) until the birds were deeply anesthetized, at which time they were euthanized by severing the spinal cord.

### Animal Ethics Approval

Isoflurane and urethane recordings were carried out in accordance with the recommendations of the La Trobe University Animal Ethics Committee. These protocols were approved by the Animal Ethics Committee at La Trobe University. Methods for the natural sleep recordings were approved by the Government of Upper Bavaria (Germany) and adhered to the NIH standards regarding the care and use of animals in research.

### Data Analyses

#### Scoring

Natural sleep was scored in 4 s epochs for the presence or absence of SWS. An epoch was categorized as SWS when it showed high amplitude, slow waves along with behavioral inactivity, as verified by inspection of video recordings. Isoflurane EEG data was scored in 2 s epochs for the presence of slow waves, or a mixed epoch in which an isoelectric EEG made up more than 0.5 s, but less than 1.0 s, of the epoch. Epochs lacking slow waves, and mixed epochs, were excluded from all analyses. A shorter epoch duration was used for scoring the isoflurane data since periods of suppression under isoflurane were short in duration. Under urethane anesthesia, at all dosages, the presence of slow waves was constant, thus scoring was not necessary. For the purposes of the multi-taper spectral analyses (see below), urethane data was analyzed using 2 s epochs.

#### Power Analysis

Power analyses were carried out on all EEG recordings for quantitative comparison. Spectral power density was calculated following a multi-taper approach (Thomson, 1982; Prerau et al., 2017), using the ‘multi_taper_psd’ function of the Python (version 3.6) Nitime toolbox (version 0.7^2^) with a bandwidth parameter of 2 Hz and 2 s epochs (window size 400 samples). Paired, two-tailed *t*-tests were performed comparing frequency bins for each recording channel among natural sleep, isoflurane, and urethane data.

#### Interhemispheric Asymmetry Analysis

During the scoring of isoflurane signals, burst suppression was observed to occur both bihemispherically and unihemispherically. Consequently, an interhemispheric asymmetry index (L-R/L+R; L and *R* = 0.78–3.90 Hz power density for the left and right hemisphere of each brain region, respectively; following Rattenborg et al., 2016) was calculated for all isoflurane recordings. Briefly, EEG data were fast Fourier transformed in 0.78 Hz bins, applied to Hamming-windowed data, using REMLogic 3.4 (Embla, Broomfield, CO, United States). General linear mixed-effects models were fitted for each variable using Systat (SYSTAT 13.2; Systat Software, Inc.). Models were structured with bird identity as a random factor, isoflurane level as a fixed factor, and the asymmetry index value (arcsine square root transformed to meet the assumptions underlying the mixed model) as the dependent variable.

#### Coherence

Average coherence values were calculated using the coherence function in the scipy computing package (version 1.0.0) in Python 3 (version 3.6.4), using Welch’s method based on Hann windows of 200 samples, and 100 samples overlap. Coherence values were calculated between brain regions within a hemisphere (e.g., between the hyperpallia and mesopallia within the left hemisphere) and between corresponding regions across hemispheres (e.g., between the left and right mesopallia). Coherence was averaged for frequency bins from 0.5 to 4.5 Hz. For anesthetized recordings, coherence was calculated for periods containing only slow waves. For natural sleep recordings, coherence was calculated for all nighttime SWS events. Coherence values for each channel comparison were compared using paired, two-tailed *t*-tests.

The raw data supporting the conclusions of this manuscript will be made available by the authors, without undue reservation, to any qualified researcher.

## Results

**FIGURE 1:**
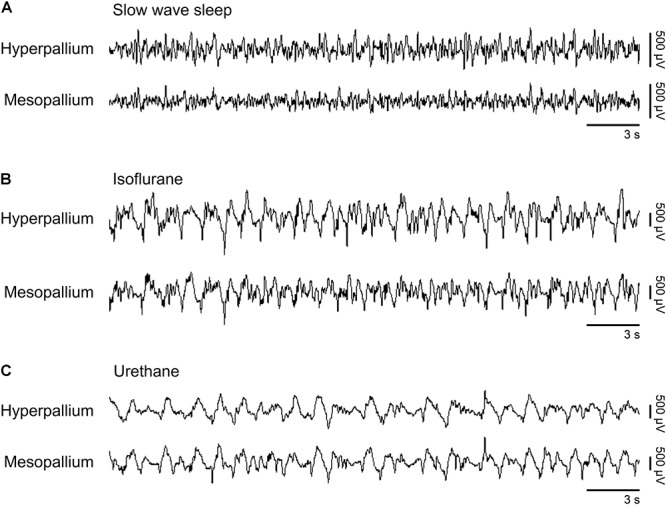
Representative EEG patterns from the right hemisphere during **(A)** natural slow wave sleep, **(B)** isoflurane, and **(C)** urethane anesthesia. High-pass filter set at 0.5 Hz; low-pass filter at 25 Hz. Horizontal bars below traces correspond to 3 s of EEG (total trace duration is 30 s). Vertical bars to the right represent voltage scale. Isoflurane and urethane examples are scaled the same, whereas the slow wave sleep example is scaled 2.5× that of the anesthetic traces.

Slow waves occurring during natural SWS were visually similar to slow waves occurring under isoflurane and urethane (Figure 1). However, slow wave amplitude was higher under both anesthetics than during SWS. Next, we proceed by comparing power spectra of SWS to that of isoflurane- and urethane-induced brain activity, followed by a direct comparison of the two anesthetics.

**FIGURE 2:**
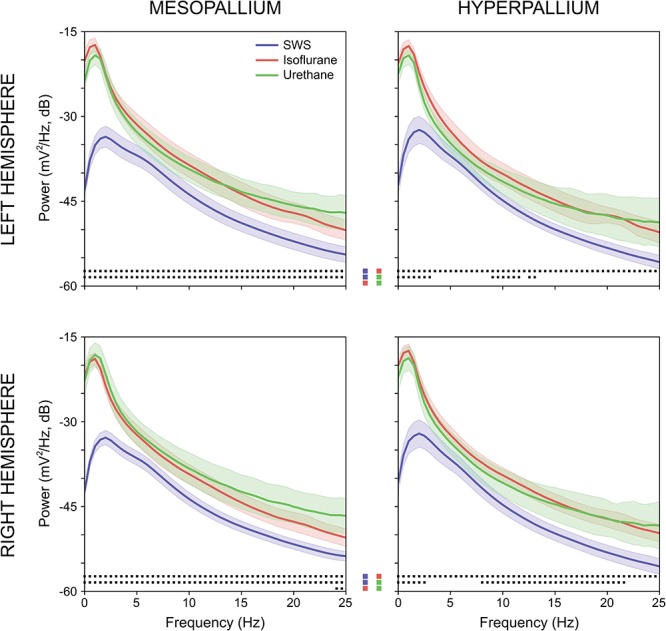
Comparison of mean absolute power for the left and right mesopallia and hyperpallia during natural slow wave sleep (SWS) (blue), isoflurane (red), and urethane (green). Shading reflects confidence intervals. Squares at the bottom of each panel denote statistically significant differences (between 0.5 Hz frequency bins) between recordings. Colored squares in the center of the figure reflect the specific statistical comparisons; i.e., the top row compares SWS to isoflurane, followed by slow wave sleep to urethane, and lastly isoflurane and urethane.

**FIGURE 3:**
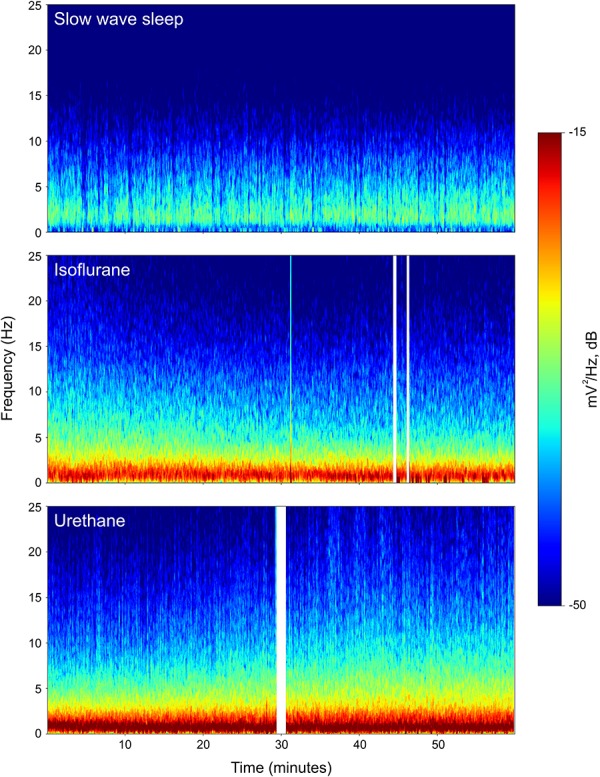
Representative multi-taper spectrograms from the right hyperpallia for 1 h of sleep, isoflurane, and urethane. Different colors reflect different power values, with reds denoting the highest power, and blues, the lowest. White vertical bars in the isoflurane and urethane panels reflect omitted data arising from experimenter-induced artifacts. Data from the same individual pigeon is shown for the isoflurane and urethane spectra.

Power associated with SWS, isoflurane, and urethane was concentrated < 3 Hz, and then declined (Figure 2). Despite this broad similarity, spectral differences were evident. Peak power under isoflurane and urethane occurred 1–1.5 Hz, but 2–2.5 Hz for SWS. Further, power during SWS was consistently lower, across all frequencies and brain regions examined, than under isoflurane. Similarly, SWS-related power was lower than that of urethane for all frequencies examined in the mesopallium. A similar pattern was observed in the hyperpallium; however, significance was more intermittent, perhaps owing to increased hyperpallial variability under urethane. These mean reductions in power can also be visualized with multi-taper spectrograms (Figure 3). In contrast to these differences between SWS, and isoflurane and urethane, these two anesthetics were more similar to one another than either was to natural sleep. EEG power under isoflurane and urethane was not significantly different for any frequency bin, of any brain region, with a minor exception of 24–25 Hz activity in the right mesopallium.

### Interhemispheric Asymmetry

**FIGURE 4:**
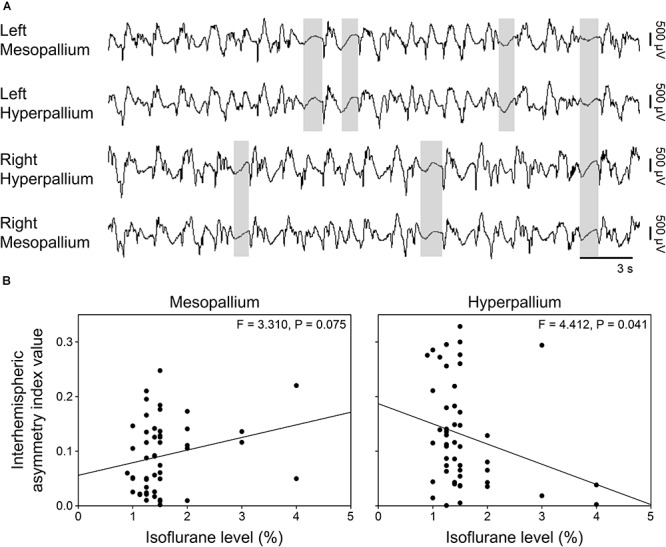
Asymmetric burst suppression under isoflurane. **(A)** Example of unihemispheric and bihemispheric suppression occurring under isoflurane. Suppression periods are highlighted by transparent gray rectangles. EEG channels are high-pass filtered at 0.5 Hz, low-pass filtered at 25 Hz, and scaled similarly. Traces are 30 s; the horizonal bar corresponds to 3 s of EEG. Vertical bars to the right represent 500 μV of EEG amplitude. **(B)** Plots of mean interhemispheric asymmetry index values per isoflurane level for the mesopallium and hyperpallium. Each datum represents the average asymmetry index value for a given period sample at the indicated isoflurane level.

Burst suppression occurred both bi- and unihemispherically under isoflurane anesthesia (Figure 4A). Interhemispheric asymmetry indices were calculated for each corresponding channel pair to look for local differences in asymmetry. Interestingly, asymmetry index values significantly decreased in the hyperpallium as isoflurane levels increased, while asymmetry in the mesopallium showed the opposite trend (Figure 4B).

### Coherence

Coherence analyses look at the similarity between two signals (e.g., the wave forms recorded from left and right hyperpallium or left hyper- and mesopallia). This type of analysis has the potential to reveal functional connectivity between brain regions by examining correlations among signals as a function of frequency. The coherence analysis was restricted to the 0.5–4.5 Hz frequency band to focus on the coherence of EEG slow waves. Average coherence was calculated over burst periods for isoflurane recordings, and over the entire recording period for urethane recordings. For natural sleep recordings, average coherence was calculated for periods of artifact-free SWS. Intrahemispheric coherence was similar in the left hemisphere for SWS, isoflurane, and urethane (Table 1). Intrahemispheric coherence between the hyperpallium and mesopallium in the right hemisphere was similar between SWS and urethane, as well as between SWS and isoflurane. Interhemispheric coherence was also similar between hyperpallial recording sites between SWS, and isoflurane and urethane. Interhemispheric coherence was significantly different between mesopallial recording sites between SWS and urethane, and tended to be higher under isoflurane.

**Table 1:** Summary of intra- and interhemispheric coherence values for various recording conditions.

	Left intrahemispheric	Right intrahemispheric	Interhemispheric hyperpallium	Interhemispheric mesopallium
Isoflurane	0.66 ± 0.02	0.71 ± 0.05	0.13 ± 0.03	0.08 ± 0.03
Urethane	0.73 ± 0.06	0.72 ± 0.08	0.20 ± 0.05	0.04 ± 0.01^∗^
SWS	0.62 ± 0.04	0.61 ± 0.05	0.12 ± 0.03	0.01 ± 0.003


*Recording conditions are indicated in the first column; sites being compared are indicated along the top row. ^∗^*p* < 0.01.*

## Discussion

### Spectral Comparison of SWS and Anesthesia

The spectral properties of electrophysiological patterns in anesthetized birds had not previously been investigated systematically. In this study, we investigated the spectral properties of slow waves occurring under both isoflurane and urethane, for comparison with those during SWS. In all recording conditions, the brain generated high amplitude, slow waves, associated with peak power density below 3 Hz. Nonetheless, power density was elevated under anesthesia when compared to SWS for all frequencies examined. This elevation, together with the concentration of power in the slowest frequencies under anesthesia, could indicate that these anesthetics are acting on components of sleep-promoting pathways that contribute to lower frequency signals. Accordingly, isoflurane acts on pathways involved in the generation of natural sleep in mammals (Moore et al., 2012) and flies (Cohen et al., 2016; Zalucki and van Swinderen, 2016), and the slow waves induced by isoflurane appear to relieve the need for SWS as if spontaneous SWS had occurred (Nelson et al., 2010). Interestingly, however, under ketamine-xylazine, cats exhibit lower power in lower frequency ranges (0.1–4 Hz and 8–14 Hz) as compared to SWS (Chauvette et al., 2011). Additional studies are needed to determine whether the differences in power spectra observed in cats under ketamine-xylazine, and pigeons under isoflurane and urethane, are due to the use of different anesthetics, or to the species examined.

Interestingly, coherence varied between brain hemispheres in pigeons. Interhemispheric coherence between mesopallial recording sites was higher under urethane anesthesia, and showed a trend for higher coherence for burst periods under isoflurane anesthesia compared to SWS. Meanwhile, interhemispheric coherence did not vary between hyperpallial recording sites for either anesthetic. In mammals, anesthetic-induced states are generally associated with higher coherence of brainwaves in the 0.1–4 Hz frequency range (Chauvette et al., 2011). Interhemispheric asymmetry also showed variability in response to differing doses of isoflurane between the mesopallium and hyperpallium. The cause and significance of these differences are unclear. It is also unclear why brainwave patterns under anesthesia didn’t show increased synchrony, as observed in mammals. Perhaps differences in interconnectivity (Shanahan et al., 2013), functional lateralization of the avian brain (Rogers, 1980, 2008), and/or absence of a corpus callosum (Cuénod, 1974) could affect the synchrony of brainwave patterns in birds, and thus account for the variation in coherence and interhemispheric asymmetry between brain regions, and between mammals and birds.

### Interhemispheric Asymmetry Under Isoflurane Anesthesia

Periods of suppression under isoflurane occurred both bihemispherically and unihemispherically. Birds also engage in unihemispheric SWS, however, the anatomical correlates allowing the two hemispheres to sleep independently remain unclear (Ball et al., 1986; Rattenborg et al., 1999, 2000; Fuchs et al., 2009; Rattenborg et al., 2016). Birds lack a corpus callosum (Cuénod, 1974), a structure connecting the cerebral hemispheres in eutherian mammals, which acts as the main route of interhemispheric communication. Interestingly, similar asymmetrical suppression patterns have been described in humans lacking a corpus callosum when anesthetized with pentobarbital (Lazar et al., 1999). In addition, EEG coherence is reduced between homologous regions of each hemisphere in humans lacking a corpus callosum (Kuks et al., 1987; Nielsen et al., 1993), and following the sagittal transection of the corpus callosum in other mammals (Berlucchi, 1966; Michel, 1972; Montplaisir et al., 1990; Mohajerani et al., 2010). Nevertheless, sleep in acallosal mammals still occurs bihemispherically (Vyazovskiy and Tobler, 2005). Furthermore, dolphins, which have a corpus callosum, albeit of a greatly diminished size (Ridgway, 1986; Tarpley and Ridgway, 1994; Lyamin et al., 2008), sleep unihemispherically and exhibit asymmetrical burst suppression under anesthesia (Howard et al., 2006). Taken together, these studies suggest that the absence, or evolutionary reduction, of the corpus callosum may allow the induction of asymmetrical burst suppression under anesthesia, whereas the ability to engage in unihemispheric sleep appears to be the result of further mechanistic or structural differences.

### Urethane-Induced SWS/REM Sleep Cycling in Birds?

Rats anesthetized with urethane show cyclic EEG patterns, similar to the cycling between SWS and REM sleep that occurs during natural sleep (Clement et al., 2008). Under urethane anesthesia, chickens cycle in a similar manner between periods with EEG slow waves and periods with EEG activation in conjunction with eye movements suggestive of REM sleep (Shibata and Kadono, 1970). However, neither the cyclic EEG pattern nor REM sleep behaviors were observed under urethane anesthesia in the pigeons in our study, even though the dose was the same as that used in chickens (Shibata and Kadono, 1970). It is unclear whether or not this is due to methodological differences (such as the rate at which the dose was administered) or differences in sensitivity to urethane between chickens and pigeons. In our study, urethane was administered in increasing amounts, whereas it was administered in one large dose at the beginning of the recordings in chickens. This difference should be investigated further, as pigeons are a commonly used bird in laboratory-based studies, and the ability to induce both SWS and REM sleep with an anesthetic would be of value to those interested in certain aspects of REM sleep neurophysiology.

### Future Directions

Further investigation of anesthetic-induced states and the pathways by which they exert their effects in birds could shed light on natural sleep-promoting pathways in birds. Furthermore, studies comparing various attributes of slow waves during natural SWS to those occurring under anesthesia in birds will be important to give greater substantiation for the use of anesthetic-induced slow waves as a model for aspects of slow waves occurring during natural SWS. Also, the absence of urethane-induced SWS/REM sleep cycling observed in this study should be further investigated to determine if this is due to methodological differences or a true difference in the pigeon’s response to urethane. The presence of asymmetric burst suppression in the EEG under isoflurane underscores the ability of the avian brain hemispheres to function independently of one another during sleep and wakefulness (Ball et al., 1986; Rattenborg et al., 1999, 2000, 2016; Fuchs et al., 2009). It would be of interest to study interhemispheric coherence of isoflurane-induced burst suppression periods in other birds, as well as mammals lacking a corpus callosum, such as marsupials and monotremes (Suárez et al., 2017).

## Conclusion

Electrophysiological patterns occurring under isoflurane and urethane anesthesia share common spectral properties with those occurring during natural sleep. Spectral power is concentrated in the lowest frequency ranges, suggesting activation of brain regions involved in the genesis of the slow components of SWS. The distribution of power density is similar between anesthetics, as well as between both anesthetics and natural SWS. These results suggest isoflurane and urethane-induced slow waves represent a good model for the investigation of low frequency aspects of slow wave sleep.

## Author Contributions

RT, LT, NR, and JL contributed to conception and design of the study. LT and JL collected the data. RT and GB performed the statistical analyses. RT wrote the first draft of the manuscript. All authors contributed to manuscript revision, read, and approved the submitted version.

## Conflict of Interest Statement

The authors declare that the research was conducted in the absence of any commercial or financial relationships that could be construed as a potential conflict of interest.

## Footnotes

1 www.plastics1.com

2 http://nipy.org/nitime/

## References

[B1] Amzica F. Steriade M. (1995). Disconnection of intracortical synaptic linkages disrupts synchronization of a slow oscillation. *J. Neurosci.* 15 4658–4677. 10.1523/JNEUROSCI.15-06-04658.1995 7790931 PMC6577695

[B2] Ball N. Weaver G. Amlaner C. J. (1986). The incidence of hemispheric sleep in birds. *Sleep Res.* 15:58.

[B3] Beckers G. J. van der Meij J. Lesku J. A. Rattenborg N. C. (2014). Plumes of neuronal activity propagate in three dimensions through the nuclear avian brain. *BMC Biol.* 12:16. 10.1186/1741-7007-12-16 24580797 PMC4015294

[B4] Berlucchi G. (1966). Electroencephalographic studies in “split brain” cats. *Electroencephalogr. Clin. Neurophysiol.* 20 348–356. 10.1016/0013-4694(66)90003-4 4143672

[B5] Chauvette S. Crochet S. Volgushev M. Timofeev I. (2011). Properties of slow oscillation during slow-wave sleep and anesthesia in cats. *J. Neurosci.* 31 14998–15008. 10.1523/JNEUROSCI.2339-11.2011 22016533 PMC3209581

[B6] Clement E. A. Richard A. Thwaites M. Ailon J. Peters S. Dickson C. T. (2008). Cyclic and sleep-like spontaneous alternations of brain state under urethane anaesthesia. *PLoS One* 3:e2004. 10.1371/journal.pone.0002004 18414674 PMC2289875

[B7] Coenen A. Prinz S. van Oijen G. Bessei W. (2008). A non-invasive technique for measuring the electroencephalogram of broiler chickens in a fast way: the ‘chicken EEG clamp’ (CHEC). *Arch. Geflügelk* 71 45–47.

[B8] Cohen D. Zalucki O. H. van Swinderen B. Tsuchiya N. (2016). Local versus global effects of isoflurane anesthesia on visual processing in the fly brain. *eNeuro* 3:ENEURO.0116-16.2016.. 10.1523/ENEURO.0116-16.2016 27517084 PMC4967815

[B9] Cuénod M. (1974). “Commissural pathways in interhemispheric transfer of visual information in the pigeon,” in *The Neuroscience Study Program*, eds Schmitt F. Worden F. (Cambridge, MA: MIT Press), 21–29.

[B10] Dewasmes G. Cohen-Adad F. Koubi H. Le Maho Y. (1985). Polygraphic and behavioral study of sleep in geese: existence of nuchal atonia during paradoxical sleep. *Physiol. Behav.* 35 67–73. 10.1016/0031-9384(85)90173-8 4059402

[B11] Fuchs T. Maury D. Moore F. R. Bingman V. P. (2009). Daytime micro-naps in a nocturnal migrant: an EEG analysis. *Biol. Lett.* 5 77–80. 10.1098/rsbl.2008.0405 18990656 PMC2657733

[B12] Gugino L. D. Chabot R. J. Prichep L. S. John E. R. Formanek V. Aglio L. S. (2001). Quantitative EEG changes associated with loss and return of consciousness in healthy adult volunteers anaesthetized with propofol or sevoflurane. *Br. J. Anaesth.* 87 421–428. 10.1093/bja/87.3.421 11517126

[B13] Howard R. S. Finneran J. J. Ridgway S. H. (2006). BIS monitoring of unihemispheric effects in dolphins. *Anesth. Anal.* 103 626–632. 10.1213/01.ane.0000231656.38488.b4 16931672

[B14] Kottler B. Bao H. Zalucki O. Imlach W. Troup M. van Alphen B. (2013). A sleep/wake circuit controls isoflurane sensitivity in Drosophila. *Curr. Biol.* 23 594–598. 10.1016/j.cub.2013.02.021 23499534

[B15] Kroeger D. Amzica F. (2007). Hypersensitivity of the anesthesia-induced comatose brain. *J. Neurosci.* 27 10597–10607. 10.1523/JNEUROSCI.3440-07.2007 17898231 PMC6673173

[B16] Kuks J. B. Vos J. E. O’Brien M. J. (1987). Coherence patterns of the infant sleep EEG in absence of the corpus callosum. *Electroencephalogr. Clin. Neurophysiol.* 66 8–14. 10.1016/0013-4694(87)90132-5 2431870

[B17] Lazar L. M. Milrod L. M. Solomon G. E. Labar D. R. (1999). Asynchronous pentobarbital-induced burst suppression with corpus callosum hemorrhage. *Clin. Neurophysiol.* 110 1036–1040. 10.1016/S1388-2457(99)00046-2 10402090

[B18] Lesku J. A. Rattenborg N. C. (2014). Avian sleep. *Curr. Biol.* 24 R12–R14. 10.1016/j.cub.2013.10.005 24405667

[B19] Lesku J. A. Roth T. C. Rattenborg N. C. Amlaner C. J. Lima S. L. (2008). Phylogenetics and the correlates of mammalian sleep: a reappraisal. *Sleep Med. Rev.* 12 229–244. 10.1016/j.smrv.2007.10.003 18403222

[B20] Lesku J. A. Vyssotski A. L. Martinez-Gonzalez D. Wilzeck C. Rattenborg N. C. (2011). Local sleep homeostasis in the avian brain: convergence of sleep function in mammals and birds? *Proc. R. Soc. B* 278 2419–2428. 10.1098/rspb.2010.2316 21208955 PMC3125620

[B21] Libourel P. A. Herrel A. (2016). Sleep in amphibians and reptiles: a review and a preliminary analysis of evolutionary patterns. *Biol. Rev.* 91 833–866. 10.1111/brv.12197 26031314

[B22] Lyamin O. I. Manger P. R. Ridgway S. H. Mukhametov L. M. Siegel J. M. (2008). Cetacean sleep: an unusual form of mammalian sleep. *Neurosci. Biobehav. Rev.* 32 1451–1484. 10.1016/j.neubiorev.2008.05.023 18602158 PMC8742503

[B23] McIlhone A. E. Beausoleil N. J. Kells N. J. Johnson C. B. Mellor D. J. (2018). Effects of halothane on the electroencephalogram of the chicken. *Vet. Med. Sci.* 4 98–105. 10.1002/vms3.91 29851306 PMC5980213

[B24] Michel F. (1972). “Sleep and waking in cats with various sagittal section of the brain,” in *Cerebral Interhemispheric Relations*, eds Cernacek J. Podivinsky F. (Bratizlava, SVK: Slovak Academy of Sciences), 83–97.

[B25] Mohajerani M. H. Chan A. W. Mohsenvand M. LeDue J. Liu R. McVea D. A. (2013). Spontaneous cortical activity alternates between motifs defined by regional axonal projections. *Nat. Neurosci.* 16 1426–1435. 10.1038/nn.3499 23974708 PMC3928052

[B26] Mohajerani M. H. McVea D. A. Fingas M. Murphy T. H. (2010). Mirrored bilateral slow-wave cortical activity within local circuits revealed by fast bihemispheric voltage-sensitive dye imaging in anesthetized and awake mice. *J. Neurosci.* 30 3745–3751. 10.1523/JNEUROSCI.6437-09.2010 20220008 PMC6632233

[B27] Montplaisir J. Nielsen T. Côté J. Boivin D. Rouleau I. Lapierre G. (1990). Interhemispheric EEG coherence before and after partial callosotomy. *Clin. Electroencephalogr.* 21 42–47. 10.1177/155005949002100114 2297948

[B28] Moore J. T. Chen J. Han B. Meng Q. C. Veasey S. C. Beck S. G. (2012). Direct activation of sleep-promoting VLPO neurons by volatile anesthetics contributes to anesthetic hypnosis. *Curr. Biol.* 22 2008–2016. 10.1016/j.cub.2012.08.042 23103189 PMC3628836

[B29] Nath R. D. Bedbrook C. N. Abrams M. J. Basinger T. Bois J. S. Prober D. A. (2017). The jellyfish Cassiopea exhibits a sleep-like state. *Curr. Biol.* 27 2984–2990. 10.1016/j.cub.2017.08.014 28943083 PMC5653286

[B30] Nelson A. B. Faraguna U. Tononi G. Cirelli C. (2010). Effects of anesthesia on the response to sleep deprivation. *Sleep* 33 1659–1667. 10.1093/sleep/33.12.1659 21120128 PMC2982736

[B31] Nielsen T. Montplaisir J. Lassonde M. (1993). Decreased interhemispheric EEG coherence during sleep in agenesis of the corpus callosum. *Eur. Neurol.* 33 173–176. 10.1159/000116928 8467828

[B32] Omond S. Ly L. M. T. Beaton R. Storm J. J. Hale M. W. Lesku J. A. (2017). Inactivity Is nycthemeral, endogenously generated, homeostatically regulated, and melatonin modulated in a free-living platyhelminth flatworm. *Sleep* 40:zsx124. 10.1093/sleep/zsx124 28958003

[B33] Ookawa T. (1967). Electroencephalographic study of the chicken telecephalon in wakefulness, sleep and anesthesia. *Acta Sch. Med. Gifu* 15 76–85.

[B34] Prerau M. J. Brown R. E. Bianchi M. T. Ellenbogen J. M. Purdon P. L. (2017). Sleep neurophysiological dynamics through the lens of multitaper spectral analysis. *Physiology* 32 60–92. 10.1152/physiol.00062.2015 27927806 PMC5343535

[B35] Rattenborg N. C. Amlaner C. J. Lima S. L. (2000). Behavioral, neurophysiological and evolutionary perspectives on unihemispheric sleep. *Neurosci. Biobehav. Rev.* 24 817–842. 10.1016/S0149-7634(00)00039-7 11118608

[B36] Rattenborg N. C. Lima S. L. Amlaner C. J. (1999). Half-awake to the risk of predation. *Nature* 397 397–398. 10.1038/17037 29667967

[B37] Rattenborg N. C. Martinez-Gonzalez D. Roth T. C. Pravosudov V. V. (2011). Hippocampal memory consolidation during sleep: a comparison of mammals and birds. *Biol. Rev.* 86 658–691. 10.1111/j.1469-185X.2010.00165.x 21070585 PMC3117012

[B38] Rattenborg N. C. Voirin B. Cruz S. M. Tisdale R. Dell’Omo G. Lipp H. P. (2016). Evidence that birds sleep in mid-flight. *Nat. Commun.* 7:12468. 10.1038/ncomms12468 27485308 PMC4976198

[B39] Ridgway S. (1986). “Physiological observations on dolphin brains,” in *Dolphin Cognition and Behavior: A Comparative Approach*, eds Thomas J. Wood F. (Hillsdale, NJ: Lawrence Erlbaum Associates), 31–59.

[B40] Rogers L. J. (1980). Lateralization in the avian brain. *Bird Behav.* 2 1–12. 10.3727/015613880791573835

[B41] Rogers L. J. (2008). Development and function of lateralization in the avian brain. *Brain Res. Bull.* 76 235–244. 10.1016/j.brainresbull.2008.02.001 18498936

[B42] Shanahan M. Bingman V. P. Shimizu T. Wild M. Güntürkün O. (2013). Large-scale network organization in the avian forebrain: a connectivity matrix and theoretical analysis. *Front. Comput. Neurosci.* 7:89. 10.3389/fncom.2013.00089 23847525 PMC3701877

[B43] Shein-Idelson M. Ondracek J. M. Liaw H. P. Reiter S. Laurent G. (2016). Slow waves, sharp waves, ripples, and REM in sleeping dragons. *Science* 352 590–595. 10.1126/science.aaf3621 27126045

[B44] Shibata M. Kadono H. (1970). Effects of urethane on electrocorticogram in the chicken. *Poult. Sci.* 49 1484–1491. 10.3382/ps.0491484 5501073

[B45] Siegel J. M. (2016). “Rapid eye movement sleep,” in *Principles and Practices of Sleep Mechanisms*, 5th Edn, eds Kryger M. Roth T. Dement W. C. (Philadelphia, PA: WB Saunders).

[B46] Steriade M. Nuñez A. Amzica F. (1993a). A novel slow (< 1 Hz) oscillation of neocortical neurons in vivo: depolarizing and hyperpolarizing components. *J. Neurosci.* 13 3252–3265. 8340806 10.1523/JNEUROSCI.13-08-03252.1993 PMC6576541

[B47] Steriade M. Nuñez A. Amzica F. (1993b). Intracellular analysis of relations between the slow ( < 1 Hz) neocortical oscillation and other sleep rhythms of the electroencephalogram. *J. Neurosci.* 13 3266–3283. 8340807 10.1523/JNEUROSCI.13-08-03266.1993 PMC6576520

[B48] Steriade M. McCormick D. A. Sejnowski T. J. (1993c). Thalamocortical oscillations in the sleeping and aroused brain. *Science* 262 679–685. 8235588 10.1126/science.8235588

[B49] Suárez R. Paolino A. Kozulin P. Fenlon L. R. Morcom L. R. Englebright R. (2017). Development of body, head and brain features in the Australian fat-tailed dunnart (*Sminthopsis crassicaudata*; Marsupialia: dasyuridae): a postnatal model of forebrain formation. *PLoS One* 12:e0184450. 10.1371/journal.pone.0184450 28880940 PMC5589244

[B50] Tarpley R. J. Ridgway S. H. (1994). Corpus callosum size in delphinid cetaceans. *Brain Behav. Evol.* 44 156–165. 10.1159/000113587 7987664

[B51] Thomson D. J. (1982). Spectrum estimation and harmonic analysis. *Proc. IEEE* 70 1055–1096. 10.1109/PROC.1982.12433

[B52] Tisdale R. K. Lesku J. A. Beckers G. J. L. Rattenborg N. C. (2018). Bird-like propagating brain activity in anesthetized Nile crocodiles. *Sleep* 41:zsy105. 10.1093/sleep/zsy105 29955880

[B53] Van Der Meij J. Beckers G. J. L. Rattenborg N. C. (2015). “Intracerebral recordings of slow wave and rapid-eye movement sleep in naturally sleeping pigeons,” in *Program No. 193.14/A103. 2-15 Neuroscience Meeting Planner*, (Washington, DC: Society for Neuroscience).

[B54] Vyazovskiy V. V. Delogu A. (2014). NREM and REM sleep: complementary roles in recovery after wakefulness. *Neuroscientist* 20 203–219. 10.1177/1073858413518152 24598308

[B55] Vyazovskiy V. V. Tobler I. (2005). Regional differences in NREM sleep slow-wave activity in mice with congenital callosal dysgenesis. *J. Sleep Res.* 14 299–304. 10.1111/j.1365-2869.2005.00456.x 16120105

[B56] Zalucki O. van Swinderen B. (2016). What is unconsciousness in a fly or a worm? A review of general anesthesia in different animal models. *Conscious. Cogn.* 44 72–88. 10.1016/j.concog.2016.06.017 27366985

